# Lung Function and Respiratory Health of Populations Living Close to Quarry Sites in Palestine: A Cross-Sectional Study

**DOI:** 10.3390/ijerph17176068

**Published:** 2020-08-20

**Authors:** Maysaa Nemer, Rita Giacaman, Abdullatif Husseini

**Affiliations:** Institute of Community and Public Health, Birzeit University, Birzeit P.O. Box 14, Palestine; rita@birzeit.edu (R.G.); abdullatif@birzeit.edu (A.H.)

**Keywords:** environmental exposure, quarry dust, respiratory disease, lung function tests, Palestine

## Abstract

Environmental exposure to dust from quarrying activities could pose health dangers to the population living nearby. This study aimed to investigate the health effects of dust exposure on people living close to quarry sites and compared them with those who live far from the quarry sites. A cross-sectional comparative study was conducted among 79 exposed participants, who lived less than 500 m away from the quarry sites, and 79 control participants who lived more than 500 m away. All participants answered a questionnaire on dust exposure at home and health effects, as well as performed a lung function test in which both reported and measured health effects were investigated. People who live in close proximity to the quarry sites reported exposure to dust at home (98%), land destruction (85%), plant leaves covered with dust (97%), and an inability to grow crops (92%). The exposed group reported significantly higher eye and nasal allergy (22% vs. 3%), eye soreness (18% vs. 1%), and dryness (17% vs. 3%), chest tightness (9% vs. 1%), and chronic cough (11% vs. 0%) compared to the control group. Lung function parameters were significantly lower among the exposed group compared to the control group; mean forced vital capacity (FVC) was 3.35 L vs. 3.71 L (*p* = 0.001), mean forced expiratory volume in the first second (FEV_1_) was 2.78 L vs. 3.17 L (*p* = 0.001). Higher levels of airway restriction were found among the exposed group. Among the exposed group, lung function parameters worsened with the increasing closeness of home to the quarry site. This study demonstrates the negative health effects of environmental dust exposure among two communities living near quarry sites in Palestine. The results highlight the importance of developing and strictly enforcing rules and regulations in Palestine to protect population health.

## 1. Introduction

The stone and marble industry is one of the most important and active industrial sectors in Palestine and contributes to about 25% of Palestine’s overall industrial revenues and 4.5% of the total Palestinian Gross National Product [1]. The total number of stone and marble facilities in the West Bank and Gaza Strip is 1124. These vary between quarries, factories, and cutting workshops [2]. Stone quarrying is a multistage process by which rock is extracted from the ground and crushed to produce aggregate, which is then screened into desired sizes for immediate use or for further processing to manufacture secondary products [2].

Despite economic importance, the stone industry has a serious negative impact on the environment at both of its types of sites: quarries and cutting workshops. The rock extraction process in quarries is the main source of dust as well as other problems, including noise, vibration, and land disturbance [3,4]. Quarrying poses a danger to workers due to injuries caused by rocks falling on the workers, accidents during the use of machinery, and dust exposure, which is the main cause of respiratory and pulmonary problems, in addition to eye problems [5,6,7].

Dust exposure in quarries and its health impact on workers has been investigated and reported both internationally and in Palestine. Several epidemiological studies worldwide suggested an association between respiratory impairment and occupational exposure to dust [8]. A high prevalence of silicosis, asthma, and adverse respiratory symptoms like cough, chest pain, and dyspnea have been reported among workers engaged in quarrying [7,9,10]. Considerable lung function impairments have been reported in quarry workers [11,12]. Studies of stone cutting workers in Palestine showed that the workers suffered from several respiratory symptoms including chronic cough, sputum production, recurrent rhinitis and shortness of breath, and had a significant deterioration in lung function [5,6].

Solid materials in the form of smoke, dust, and vapor generated during quarrying can usually suspend over a long distance in the air, and particulate matter in the air is transported from the generation point to other far areas [13]. If the quarries are located in places where there is a living population, people living in the area will also be exposed to dust. Environmental exposure to dust has been raised as an important issue to consider among populations living close to quarries in different areas around the world [13,14]. Previous studies found that people residing close to quarry sites have a higher prevalence of respiratory symptoms compared to those not exposed to quarry dust [15]. Specific reported adverse health effects by people who reside nearby quarry sites include nasal infection, cough, and asthma [13,16]. Additionally, a study investigated how the ecosystem and residents were possibly affected by nearby quarry activity found out that the frequency of certain symptoms such as cough, sneezing, and asthma, and illnesses have increased after quarry activities in the area began [17].

Exposure to quarry dust has been associated with deterioration in lung function among the quarry or mining workers [18,19,20]. Previous studies have shown that quarry and stone cutting workers had lower forced vital capacity (FVC), forced expiratory volume in the first second (FEV_1_), and FVC/FEV_1_ compared to unexposed control groups, in China [21], Libya [19], and Palestine [5,6]. Only one study from Nigeria has investigated lung function among a mixed group of workers and residents who live near quarry sites compared to a control group, which found lower lung function parameters among the workers and nearby residents compared to the control group [22].

In Palestine, there was only one study that investigated the overall environmental impacts of stone quarry work in Jammain village located in the north of the West Bank. This study found high concentrations of dust particles in the surrounding area of quarry sites, and a high prevalence of reported symptoms among the nearby population, including cough, dyspnea, nasal inflammation, as well as hearing impairment. Asthma was also reported among approximately 30% of the respondents. Approximately 75% of the declared sample reported that they suffered from noise pollution as a result of quarry activities [23].

Although previous research, in Palestine and worldwide, showed that populations living near quarry sites are exposed to dust and suffer from adverse health effects, no previous research has measured the lung function of such populations in comparison with those who live far away from quarry sites. Therefore, this study aimed to investigate the health effects of dust exposure on people living close to quarry sites and compare them with those who live far from the quarry sites in Birzeit, a town community located in the central West Bank.

## 2. Methods

### 2.1. Study Design, Site, and Population

A cross-sectional comparative study was conducted between September 2019 and January 2020. The study was conducted in Birzeit, a Palestinian town in the Ramallah Governorate located 7.5 km north of Ramallah City. It has about 7000 inhabitants [24]. The study population consisted of two groups: the exposed group, which included household members who live in houses next to quarry sites by 0–500 m, and the control group, which included household members living in the same town, whose houses are more than 500 m away from the quarry sites. It has been found that, at a distance of more than 500 m, the concentration of suspended particulate matter is significantly reduced [23], which will, therefore, reduce the dust exposure. Thus, the houses of the control group members were located in areas that are more than 500 m away from the quarry sites.

### 2.2. Study Sample

According to the information collected from the Birzeit Municipality, there is a total of three main quarrying and stone cutting sites in Birzeit town. We selected a random sample of around 100 household members who live near each quarry site within a circle of 500 m diameter (exposed group), and a random sample of around 100 household members who live at more than 500 m away from the quarry site, i.e., populations out of the 500 m radius were selected (control group). The three available quarry sites inside the boundaries of Birzeit town were located on the community map (Q1, Q2, Q3). A circle with the quarry at the center and a radius of 500 m was drawn around each quarry site. All the houses within the area of the circle were included in the exposed group. All the houses outside the area of the circle were included in the control group. The houses were randomly selected from each group until we reached the needed number of houses from each group (30–40 houses) depending on the number of people available in each house.

Inclusion criteria were considered to be all household members who have been living in the house for at least one year, and are 18 years old or above. Exclusion criteria included: any household member who has lived for less than a year in the house, and who is below the age of 18 years.

**Figure 1:**
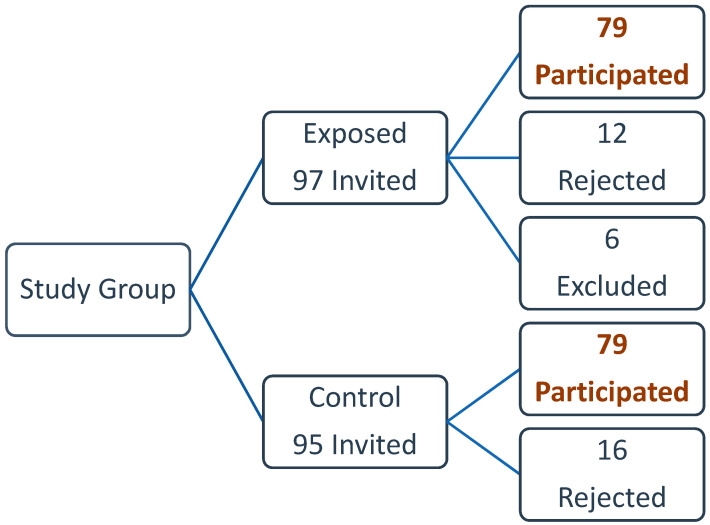
Study population and Sample.

A total of 192 participants were invited to participate. Of those, 158 agreed to participate, 28 refused to participate, and six have lived in the house for less than a year, thus excluded from the study. Three participants refused to perform the lung function test. This made the total number of participants 158: 79 living in households close to the quarry sites (exposed group), and 79 away from them (control group) (Figure 1).

The number of participants who came from the same household ranged from 1–3 participants, with only one household that included three participants, 31 households included two participants each, and the rest of the participants, 93, each came from one household. Having more than one observation from the same household, as in our case, might raise the issue of clustering, as we will have two levels: the participants (level 1), and the household (level 2), known as naturally occurring clusters. However, as we have a large number of clusters (households), with a very small number of participants in each cluster, a situation known as “sparsely clustered data” [25], we have not used a multi-level model for analysis [25,26].

### 2.3. Questionnaire

The questionnaire was administered face to face and included demographic and socioeconomic information (age, sex, level of education, type of work), smoking habits and history, location of the house from the quarry site (distance), frequency of dust exposure, years of living in the area, the year when the quarry was established nearby, general health conditions, specific respiratory symptoms (used to detect asthma and chronic obstructive pulmonary disease (COPD), and adapted from an internationally standardized respiratory questionnaire) [27], allergic symptoms, eye and nose symptoms, and auditory symptoms related to noise exposure.

### 2.4. Lung Function Test (Spirometry)

Lung function tests (spirometry) have been widely used to detect deterioration in the respiratory function among occupational and non-occupational groups exposed to dust [19,22,28,29,30]. The main lung function parameters are forced vital capacity (FVC) and forced expiratory volume in the first second (FEV_1_) [31,32]. Normal spirometry means that all measured parameters (FVC, FEV_1_, and FEV_1_/FVC) are 80% or higher of the expected values compared to their sex, age, height, weight, smoking, and ethnicity [32,33,34]. An obstructive pattern means that FEV_1_ is lower than 80% of the predicted value, FVC is reduced but to a lesser extent than the FEV_1_, and the ratio is also reduced to a lower than 0.7 [32,33]. A restrictive pattern means that both FEV_1_ and FVC are reduced to lower than 80% of the predicted value, and the ratio is normal (above 0.7) [32,33].

Lung function tests were performed by a trained researcher using a portable Spirometer (MicroLab, Vyaire Medical GmbH, Germany). Measurements were carried out according to standard protocols of the American Thoracic Society (ATS) guidelines [31]. Participants were given enough time to understand the test procedure and provide the required flows. During the test, participants were seated, with the lips firmly applied around the disposable mouthpiece and using a nose clip. Three reproducible attempts were allowed for each participant, and the best flow was automatically selected by the spirometer.

### 2.5. Data Collection

The data collection was conducted by two fieldworkers, who visited the houses, invited the inhabitants to participate, and explained the aim and the process of participation in the study. The fieldwork was conducted in the period from September 2019 to January 2020. After taking informed written consent, the participants were interviewed and asked the questions of the questionnaire and then asked to perform the lung function test.

### 2.6. Statistical Analysis

Descriptive statistics were performed to compare results between the two groups to make sure that they are comparable in terms of demographic and socioeconomic factors. Means and standard deviations were used to present continuous variables, and frequencies were used to present dichotomous variables. Our outcome variables were specific respiratory symptoms, asthma, nasal and eye infections, and lung function parameters (FEV_1_, FVC, and the FEV_1_/FVC ratio). Data on dust exposure at home, environmental effects of quarry activities on the residents, and air pollution from quarry activities were analyzed for the exposed group separately. Comparisons between the two study groups were performed by an independent t-test for continuous variables, χ^2^ test for dichotomous variables, and linear regression was used when comparing the lung function parameters between the two groups, to adjust for sex, age, height, weight, and smoking between them, factors that are known to affect the lung function results. All statistical analyses were performed using the SPSS V.24.0 software for Windows. All *p* values were two-sided, and a *p*-value < 0.05 was considered significant.

## 3. Results

There were variations in the exposed and control groups by sex (39 men vs. 8 men and 40 women vs. 71 women, respectively), mean age (37 years vs. 32 years, respectively), smoking status (12 smokers vs. 2 smokers, respectively), and employment status (28 unemployed vs. 47, respectively). These two groups were similar in the mean number of years of education (Table 1). None of the participants is a quarry or stone cutting worker.

### 3.1. Environmental Effects of Quarrying Activities on the Population Living Nearby

The mean distance between the houses of the exposed group and the quarry sites was 247.2 m, with a range of 50–500 m. The residents in this group have lived in the area for 9.98 years on average, with a range of 3–33 years. The main environmental effects of the quarrying activities which were reported by the exposed group included land destruction (85%) which means a change in the features of the land and the landscape, inability to grow crops in the surrounding area (92%), and that there is need for land restoration (87%). Only 33% of respondents reported that there were heaps of waste and holes filled with water around the quarry sites, and 8% reported that the farmlands around the quarry sites were filled with water coming from the quarry work. The residents reported that the landscape has changed over the past ten years due to the quarrying activities in the area. Table 2 shows that 67% described the landscape to be vegetative or green 10 years ago, while 20% described it to be vegetative at the time of the study. Additionally, 11% described the landscape to be bare 10 years ago, while 51% described it to be bare at the time of the study.

**Figure 2:**
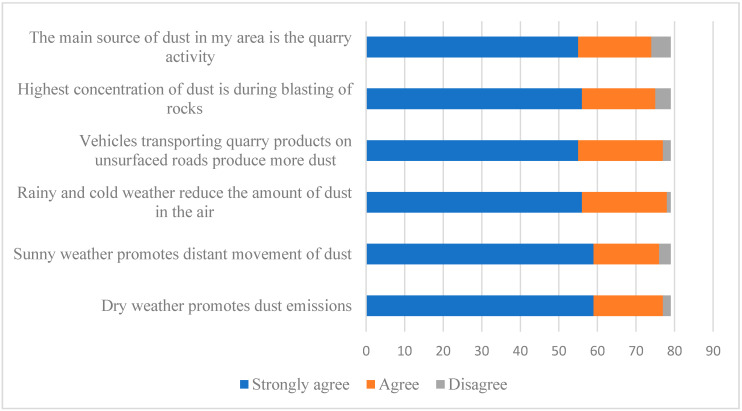
Dust and air pollution from quarry activities as described by the population close to the quarry sites (*n* = 79).

**Figure 3:**
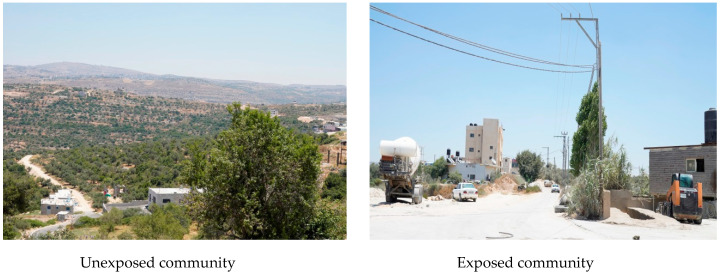
Pictures of exposed and unexposed communities at Birzeit Town.

One of the most adverse effects of quarrying activities, as described by the residents, was dust. Almost all the participants (98%) reported that dust settles on their house roofing, surfaces, and clothing at home; 97% reported that the leaves of plants and crops around the house are covered with dust, and 96% reported that plants do not grow well when the dust covers their leaves. On the effects of dust on vision, only 20% of the participants reported that dust prevents them from seeing things a distance away. Figure 2 shows that most participants reported that the main source of dust in their area was the quarry activity and that the dust movement increased during sunny and dry weather. In addition, they reported that vehicles that transport quarry products produce more dust in the area. Participants also reported that quarrying activities are the main source of noise in the area (75%), but few reported that this had affected their hearing (14%). Almost all the participants reported that there are no vibration effects of the quarrying activities, with only one participant reported that quarrying caused cracks of their building due to vibration. Figure 3 includes a picture of one exposed and another unexposed community at Birzeit Town, showing the effects of dust on the exposed community on the nearby houses and the green areas, compared to the unexposed community with more green areas and less amount of dust.

### 3.2. Reported Symptoms and Diseases

The exposed group reported more rhinitis (13% vs. 3%), eye or nasal allergy (22% vs. 3%), and irritant eye symptoms including tearing (19% vs. 1%), soreness (18% vs. 1%), and dryness of the eye (17% vs. 3%) compared to the control group, Table 3. Asthma and bronchitis were reported by two participants in the exposed group and none in the control group. For the respiratory symptoms, chest tightness, shortness of breath and chronic cough were significantly higher among the exposed group compared to the control group; 9% vs. 1%, 25% vs. 1%, and 11% vs. 0%, respectively, Table 3.

### 3.3. Lung Function

The mean values of lung function parameters for the exposed group were lower than those for the control group. When the two groups were compared using a linear regression model, adjusting for sex, age, height, weight, and smoking, the exposed group had significantly lower values of FEV_1_, FVC, and FEV_1_/FVC compared with the control group, Table 4.

**Figure 4:**
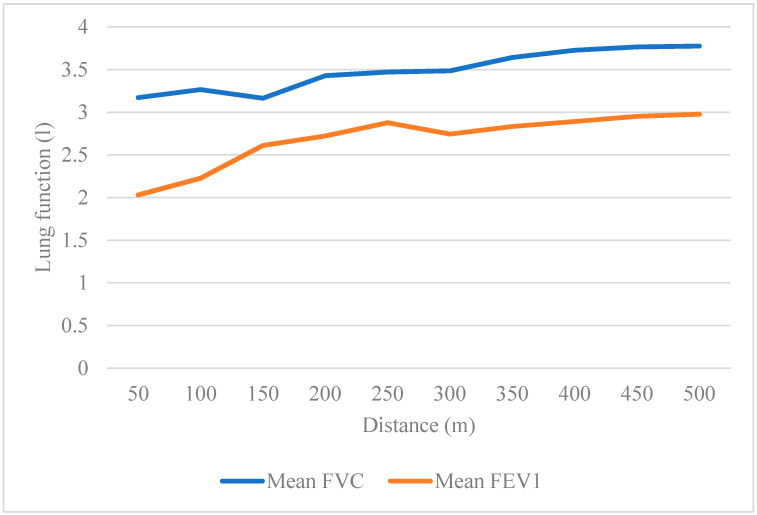
Lung function parameters among the exposed group according to the distance from the quarry site.

Normal spirometry (normal FVC, FEV_1,_ and FEV_1_/FVC ratio) was found among 43 exposed participants compared with 71 control participants. Only one exposed participant showed mild obstruction (low FEV_1_, normal FVC, low ratio), and one showed moderate obstruction (very low FEV_1_, normal FVC, very low ratio). Mild restriction (low FVC, normal FEV_1,_ and normal ratio) was found among 15 exposed compared to 7 control participants. Moderate restriction (low FVC, low FEV_1,_ and normal ratio) was found among 13 exposed compared to one control participant. Severe restriction (very low FVC, low FEV_1_, and normal ratio) was found among the three exposed participants. The participants in the exposed group differed in lung function parameters according to the distance from the quarry site. The closer to the quarry site, the lower FVC, and FEV_1_ they had, Figure 4.

### 3.4. Mitigation Measures as Agreed by the Participants

Participants in the two groups were asked about the most important measures for mitigation of adverse effects of the quarrying activities on the environment and the living populations. The main measures agreed on were: establishing barriers around the quarry sites, water should be used when cutting and on the road when transporting the quarry products to decrease the amount of dust, developers should not place residential areas close to quarrying zones, a license should not be given to quarries close to residential places, and violators of rules and regulations should be prosecuted.

## 4. Discussion

This study showed that the population living in close proximity (50–500 m) to quarry sites in Birzeit town are exposed to dust in their households, with 69% reporting that the main source of dust in their area was the quarry activities, and with the dust increasing in dry and sunny weather. Our study confirms what was reported in a case study in another Palestinian village in the north of the West Bank, Jammain village, where 70% of the living populations in the area close to the quarry sites were suffering from the dusty environment with dust increasing during the summer season [23]. Additionally, in a study in Hebron, a southern West Bank Palestinian city, it has been reported that the quarrying and stone cutting activities have an adverse impact on the environment and populations, mainly affecting air quality, surface, and groundwater, and contaminating agricultural soil [35]. Our study participants also reported that quarrying activities caused land destruction and the inability to grow crops. Other studies in the region found similar results. A case study conducted in Jordan found that stone cutting activities were a main source of contamination of the water and soil in the area, as well as being a main source of noise level [4].

This study also showed that people who live close to quarry sites (exposed group) reported significantly higher respiratory, eye and nasal symptoms compared to people who live far from the quarry sites (control group). Our results indicate that living in close proximity to quarry sites, which is a main source of dust, is a potential factor for increasing the prevalence of eye and respiratory symptoms. It has been found that dust is one of the most invasive and potentially irritating sources for the eyes and respiratory system [14,15,36]. Research has shown that dust concentration, deposition rates, and potential impacts tend to decrease rapidly away from the dust source [3,23]. This explains why our control participants, who live more than 500 m away from the quarry sites, have reported significantly lower symptoms. The study from Jammain village has shown that the main reported health effects among the populations living close to the quarry sites were nasal inflammation, cough, and hearing impairment [23]. Although several studies in Palestine and the region have investigated the environmental effects of the quarrying activities in terms of water, air, and soil pollution, there is a limited number of studies that investigated the health effects among the populations who live nearby. Studies from India found silicosis and other respiratory inflammatory diseases among close populations to stone mining [14], while a study conducted in Puerto Rico found an elevated prevalence of bronchitis and nasal allergy among the communities who live close to quarry sites compared to others who live far from them [15].

Lung function parameters were significantly lower for the exposed group compared with the control group, even after adjustment for sex, age, height, weight, and smoking. The lung function parameters showed that 43 out of the 76 exposed participants had normal spirometry, while almost all of the control participants (71 out of 79) had normal spirometry. Obstructive lung function impairment was found among two out of the 76 exposed participants, while restrictive impairment was found among 31 out of the 76 exposed participants. Among the control participants, none showed obstruction, and eight out of 79 showed restriction. Patterns of lung function among people exposed to dust have been mostly reported as obstruction or a combination of obstruction and restriction [28,37,38]. The obstructive pattern indicates a disease caused by the airway to be narrowed or blocked, making it difficult to exhale the air completely, as in asthma and COPD, while restrictive pattern indicates a disorder that makes it difficult to fill the lung completely with air because of interstitial lung problem such as lung fibrosis [39]. Two studies in Palestine which measured lung function for quarry workers found a combination of both obstruction and restriction [5,6]. One of those studies found more restriction among the ones with longer years of exposure [6]. Research studies indicated that the chemical content of the stone could be the main cause of respiratory diseases, such as silica, which was found to be a main causing agent of obstructive and restrictive lung diseases [11,37]. The analysis of the stone from quarries in Palestine has shown that it is mainly composed of calcium carbonate (CaCO_3_) from mining limestone, as well as silicon dioxide (SiO_2_), also known as silica or quartz [40,41]. Studies found that dust particles, which disperse in the air and are easily inhaled, decrease as it travels to long-distance [13,14]. This explains our findings among the exposed group, which showed that the lung function parameters are lower among the participants who live closer to the quarry sites.

Chronic exposure to dust has been shown to cause deterioration in lung function among several groups of working populations [42,43,44]. Several studies worldwide, including Palestine, have shown that quarry workers had lower lung function than unexposed control groups, and it was lower among the workers with longer duration of work in quarries [6,9,19,21,30,45]. Lower lung function parameters than the expected values generally indicate the possibility of chronic respiratory and lung disease [32]. As the exposed group showed lower lung function parameters than the control group, indicating that people living close to the quarry sites have a stronger possibility of developing lung disease. Previous studies that measured the lung function of people exposed to dust were only conducted among quarry or mining workers [9,11,19,20,30]. Only one study, conducted in Nigeria, assessed lung function among a mixed group of workers and residents who live near quarry sites compared to a control group [22]. The Nigerian study’s findings, although including a mixture of workers and residents, were similar to ours, as it found that workers and exposed residents had lower lung function parameters than the unexposed control group [22].

The size class and concentration of the particulate matter released from the quarrying activities in the environment have an effect on the type and extent of the adverse respiratory health effects [46]. Coarse particles larger than 10 micrometers in diameter are usually filtered in the nose and throat, thus not causing significant health problems, while fine particles (1–10 micrometers), as in quarry dust particles may have larger adverse health effects as they can reach the bronchi and cause bronchitis [46,47,48], as reported by some of the exposed participants. Very small particles of less than one micrometer could reach the alveoli [49]. This is also reflected in the lung function impairment since particles of 1–10 micrometers are known to cause more restrictive lung function impairment compared to those smaller than one micrometer, which will result in a more obstructive pattern [50,51].

As far as the authors are aware, this is the first study that investigated both reported and measured respiratory effects of environmental exposure to quarry dust among living populations in Palestine. Using both reported and measured health outcomes helped to understand the extent of the health effects and to reduce recall bias. Additionally, having a control group of the unexposed population, who lived far from the quarry sites but in the same town, helped to compare the health conditions as a result of close quarry dust exposure. However, this study has some limitations. First, the sample distribution that we had resulted in significant differences between the two groups of participants in sex, age, and smoking could have a direct effect on the compared health effects, especially lung function. The reason why the exposed group included a larger number of men than the control group is likely due to the availability of men at the house at the time of the interviews since most of the control group participants were employees, while the exposed group were workers in small workshops with more flexible time and availability at home. Additionally, the control group had a larger number of women, who were mostly unemployed and staying at home most of the time. This also explains the low number of smokers in the control group which constitutes the largest number of women among the participants, who were mostly non-smokers. To reduce the effect of these differences, they were adjusted for in the analysis when comparing lung function. Another limitation of the study is the lack of quantitative sampling of the dust using environmental samplers, which could have added a very important angle of the investigation, in terms of the concentration of the particulate matter and the size class of them. As it is known that the health effects of dust exposure depend on these factors.

## 5. Conclusions

The present study has investigated the environmental effects of quarrying activities on the populations living nearby and compared the respiratory health status of those populations with a control group of the population who lives at more than 500 m away from quarry sites. To the best of our knowledge, this is the first study in Palestine that assessed both reported and measured respiratory health effects among people living close to quarry sites. The study showed that the population that lives close to quarrying activities is exposed to a harmful amount of dust, as they reported having more respiratory and eye symptoms, and had increased levels of lung function impairment as compared with the unexposed population at the same town. The results of the study highlight the importance of developing and strictly enforcing rules and regulations in Palestine to protect population health, especially those related to the locations of quarries and the need to establish a system inside the quarry locations to reduce the amount of emitted dust in the surrounding environment.

## Figures and Tables

**Table 1:** General characteristics of the two study groups.

Variable	Exposed (*n* = 79)	Control (*n* = 79)	*p*-Value
Age *	36.76 (11.4)	31.92 (7.1)	0.004
Sex ^†^			
Men	39 (49.4)	8 (10.1)	0.001
Women	40 (50.6)	71 (89.9)	0.001
Years of education *	13.00 (1.9)	13.22 (1.8)	0.8
Occupation ^†^			
Office jobs	11 (13.9)	12 (15.2)	0.5
Industries	33 (41.8)	10 (12.9)	0.04
Education	7 (8.9)	10 (12.9)	0.07
Not working	28 (35.4)	47 (59.5)	0.05
Smoking ^†,^ⁿ	12 (15.2)	2 (2.5)	0.005
Height *	169.83 (9.40)	165.47 (6.93)	0.001
Weight *	72.99 (12.33)	65.72 (9.22)	0.007

Age and years of education are presented as means (SD). Sex, occupation, and smoking are presented as *n* (%). * Independent sample *t*-test, ^†^: χ^2^ test. ⁿ Current smoker.

**Table 2:** Landscape as described by the population living close to the quarry sites (*n* = 79).

Variable	Landscape in the Last 10 Years		Landscape Now	
	Number	Percentage	Number	Percentage
Vegetative	53	67.1	16	20.3
Rocky	12	15.2	15	16.5
Eroded	5	6.3	8	10.1
Bare	9	11.4	40	50.6

**Table 3:** Self-reported prevalence of general respiratory symptoms and diseases among the two study groups.

Self-Reported Symptoms and Diseases	Exposed (*n* = 79) *N* (%)	Control (*n* = 79) *N* (%)	*p*-Value
Chronic symptoms and diseases			
Asthma	2 (2.5)	0	0.49
Bronchitis	2 (2.5)	0	0.49
Rhinitis	10 (12.7)	2 (2.5)	0.001
Eye or nasal allergy	17 (21.5)	2 (2.5)	0.001
Eye tearing	15 (19)	1 (1.3)	0.001
Eye soreness	14 (17.7)	1 (1.3)	0.001
Eye dryness	13 (16.5)	2 (2.5)	0.005
Hearing difficulty	3 (3.8)	0	0.24
Respiratory symptoms			
Wheezing or whistling in the chest for during the last 12 months	5 (6.3)	1 (1.3)	0.21
Chest tightness during the last 12 months	7 (8.9)	1 (1.3)	0.06
Shortness of breath at rest in the last 12 months	14 (17.7)	0	0.001
Shortness of breath after physical activity in the last 12 months	20 (25.3)	1 (1.3)	0.001
Cough at night in the last 12 months	9 (11.4)	0	0.05
Cough first thing in the morning in the last 12 months	6 (7.6)	0	0.003

Differences between exposed and control using the χ^2^ test.

**Table 4:** Means and adjusted differences in lung function parameters between the two study groups.

Lung Function Parameters	Exposed (*n* = 79)		Control (*n* = 79)		Difference ^†^		*p*-Value
	Mean (%)	SD	Mean (%)	SD	Coefficient	95% CI	
FVC (l)	3.357 (84%)	0.543	3.713 (102%)	0.163	0.392	0.254–0.530	0.001
FEV_1_ (l)	2.783 (88%)	0.421	3.178 (108%)	0.131	0.401	0.292–0.510	0.001
FEV_1_/FVC	0.82	6.382	0.85	2.663	0.21	0.109–0.418	0.019

Results are presented as mean (% of predicted value) and SD: standard deviation. ^†^ Lung function differences, using linear regression, are adjusted for sex, age, height, weight, and smoking. FVC: forced vital capacity. FEV_1_: forced expiratory volume in the first second.
